# Physiological expression of mutated TAU impaired astrocyte activity and exacerbates β-amyloid pathology in 5xFAD mice

**DOI:** 10.1186/s12974-023-02823-9

**Published:** 2023-07-26

**Authors:** Dorit Farfara, Meital Sooliman, Limor Avrahami, Tabitha Grace Royal, Shoshik Amram, Lea Rozenstein-Tsalkovich, Dorit Trudler, Shani Blanga-Kanfi, Hagit Eldar-Finkelman, Jens Pahnke, Hanna Rosenmann, Dan Frenkel

**Affiliations:** 1 Department of Neurobiology, School of Neurobiology, Biochemistry and Biophysics, The George S. Wise Faculty of Life Sciences, Tel Aviv University, 6997801 Tel Aviv, Israel; 2 Department of Human Molecular Genetics and Biochemistry, Sackler School of Medicine, George S. Wise Faculty of Life Sciences, Tel Aviv University, 69978 Tel Aviv, Israel; 3 Sagol School of Neuroscience, Tel Aviv University, Tel Aviv, Israel; 4 Department of Neurology, The Agnes Ginges Center for Human Neurogenetics, Hadassah Hebrew University Medical Center, Jerusalem, Israel; 5 Section of Neuropathology, Translational Neurodegeneration Research and Neuropathology Lab, Department of Pathology, University of Oslo (UiO) and Oslo University Hospital (OUS), Oslo, Norway; 6 Drug Development and Chemical Biology, Lübeck Institute of Dermatology (LIED), University Medical Center Schleswig Holstein (UKSH), LIED, Lübeck, Germany; 7 Department of Pharmacology, Faculty of Medicine, University of Latvia, Riga, Latvia

**Keywords:** Tau, Beta-amyloid, 5xFAD, Mouse model, Astrocytes, Alzheimer’s disease, Tauopathy

## Abstract

**Background:**

Alzheimer’s disease (AD) is the leading cause of dementia in the world. The pathology of AD is affiliated with the elevation of both tau (τ) and β-amyloid (Aβ) pathologies. Yet, the direct link between natural τ expression on glia cell activity and Aβ remains unclear. While experiments in mouse models suggest that an increase in Aβ exacerbates τ pathology when expressed under a neuronal promoter, brain pathology from AD patients suggests an appearance of τ pathology in regions without Aβ.

**Methods:**

Here, we aimed to assess the link between τ and Aβ using a new mouse model that was generated by crossing a mouse model that expresses two human mutations of the human *MAPT* under a mouse *Tau* natural promoter with 5xFAD mice that express human mutated *APP* and *PS1* in neurons.

**Results:**

The new mouse model, called 5xFAD TAU, shows accelerated cognitive impairment at 2 months of age, increased number of Aβ depositions at 4 months and neuritic plaques at 6 months of age. An expression of human mutated TAU in astrocytes leads to a dystrophic appearance and reduces their ability to engulf Aβ, which leads to an increased brain Aβ load. Astrocytes expressing mutated human TAU showed an impairment in the expression of vascular endothelial growth factor (VEGF) that has previously been suggested to play an important role in supporting neurons.

**Conclusions:**

Our results suggest the role of τ in exacerbating Aβ pathology in addition to pointing out the potential role of astrocytes in disease progression. Further research of the crosstalk between τ and Aβ in astrocytes may increase our understanding of the role glia cells have in the pathology of AD with the aim of identifying novel therapeutic interventions to an otherwise currently incurable disease.

**Supplementary Information:**

The online version contains supplementary material available at 10.1186/s12974-023-02823-9.

## Introduction

Alzheimer’s disease (AD) is the most prevalent cause of dementia, accounting for about 60–80% of dementia cases [1]. The worldwide prevalence is estimated at approximately 35 million individuals whom are affected by the disorder and is predicted to rise to 65 million by 2030 [1]. The disease is defined by two major histopathological features: senile plaques, which are deposits of extracellular amyloid-β (Aβ) deposition and neurofibrillary tangles (NFTs) [2], which are deposits of the intracellular hyperphosphorylated microtubule-associated protein tau [τ, gene names: *MAPT* (human), *Mapt* (mouse)] [3]. Additional pathology is observed in neuritic plaques [2] that are associated with neuronal death. While mutations in *APP* were linked to increased Aβ production and accumulation, there was so far no mutation in the *MAPT* gene detected that was connected to AD [4]. Mutations in *MAPT* were affiliated with several neurological diseases among them familial frontotemporal dementia and Parkinsonism linked to chromosome 17 (FTDP-17), Pick’s disease (PiD), progressive supranuclear palsy (PSP) and corticobasal degeneration (CBD) [5].

There are many kinases, which phosphorylate τ under normal conditions. However, there are also several kinases, which are specifically associated with the pathology of AD [6]. These kinases include the glycogen synthase kinase-3 (GSK3β) [7]. GSK3β is a proline-directed serine/threonine kinase that takes part in cellular processes, signaling and its activation levels are increased in brains of AD patients [7]. It has been suggested that the Aβ peptide activates GSK3β resulting in hyperphosphorylation of τ [7].

In the brains of AD patients, NFTs are predominantly located in the cerebral cortex of the temporal and frontal lobes, the hippocampus and amygdala. Braak and Braak showed that NFT distribution patterns and severity best reflect AD clinical progression which was described in the 6 notorious stages [8]. In the normal brain, the τ protein is phosphorylated and dephosphorylated in a very specific manner at specific sites. A deviation from these distinct phosphorylation and dephosphorylation patterns could result in pathology, in which τ is present in a hyperphosphorylated state [9]. This might be one of the causes that lead to the existence of hyperphosphorylated τ in AD, in which no *MAPT* mutations are observed. Hyperphosphorylated τ is rejected from tubulin, causing the tubulin’s dissociation and axonal disruption which eventually leads to neuronal death. Furthermore, τ hyperphosphorylated molecules, now detached from tubulin, are more prone to aggregate and form NFTs [9]. Of note, tubulin plays an important role in the cytoskeleton of different types of cells and their ability to support cell homeostasis function [10]. Moreover, Aβ plaques are not deposited in any of the *MAPT* transgenic mice without a second human *APP* transgene due to the Aβ protein sequence variance between mouse and human at position 13 [11].

Hyperphosphorylated τ has also been observed in non-neuronal cells such as astrocytes [12]. While TAU is expressed in different types of brain cells, such as glia cells, its function within those cells is not clear. It has previously reported that *MAPT* is expressed in adult mouse astrocytes [13] and in human astrocytes [14] and in several neurodegenerative diseases [15]. Furthermore, it has also been previously reported that in different pathological conditions, astrocytes can propagate tau that may affect their ability to support neurons and trigger inflammatory properties [16]. Furthermore, overexpression of τ under the astrocytic promoter, GFAP, resulted in an accumulation of hyperphosphorylated τ in astrocytes in different brains leading to an impairment of the blood–brain barrier and neurodegeneration [17]. Nevertheless, the effect of mutant τ on glia cells, expressed under its native promoter, has not been tested before.

Rosenmann et al*.* created a tauopathy model, in which the human cDNA was mutated to implement two amino acid changes in the TAU protein (K257T and P301S), which are associated with FTDP-17. To result in physiological expression levels and locations, the natural mouse *Tau* promoter was used to express the mutated human TAU [18]. The combination of the two TAU mutations generates NFTs, which emerge at 6 months of age and are correlated with cognitive impairments, synaptic dysfunction and LTP deficits. Additionally, neuronal degradation was observed at 6 months of age [18]. However, the effect of the expression of mutated human TAU expressed in astrocytes cells under its endogenous promoter has never been tested before. Here, we aim to assess the impairment in TAU function/activity when it is expressed under its natural promotor and its effects on Aβ deposition, and cognition in a 5xFAD TAU mice.

## Materials and methods

### Mouse models

*MAPT*-transgenic mice (*MAPT*^*K257T/P301S*^) were developed in the Rosenmann lab [18]. These mice express a double-mutant human TAU cDNA (htau43) under the regulation of the original rodent *Tau* promoter [18]. The 5xFAD mice were received from Robert Vassar’s group [19]. The animals where crossed and bred as hemizygous for each mutation (Additional file 1: Fig. S1). For all the experiments we used male mice. All animal care and experimental use was in accordance with the Tel Aviv University guidelines and approved by the local authorities.

### Novel object recognition task (ORT)

The experiments were performed as previously described [20]. In brief, the mice were first placed individually for 5 min in an empty arena (50 × 50 × 20 cm^3^). This was followed on day 2 by a training session, during which one object was placed in the open field. The mice were then allowed to individually explore this object for 5 min, and the actual exploration time was then recorded. The ability of the mice to remember the object and to distinguish between novel and non-novel objects was then assessed by a retention test that was performed on day 3. Accordingly, each mouse was placed in the same box in which there was an object identical to the one used on day 2 (but placed at a different corner) and a different object placed in opposite corners of the box (10 cm from the corner). The mice were allowed to freely explore the arena for 5 min. The time spent exploring each of the objects was then recorded, and the extent to which the mice showed a preference for the new object, which is an indication that it remembered the old object, was then assessed by determining the ratio of the time spent at the new object to the total time spent at both objects. The objects used were 10–20 cm in height and were washed in ethanol between successive tests.

### ***Morphological analyses***

#### Immunohistochemistry of paraffin-embedded tissue

Formalin-fixed hemispheres (4% formaldehyde, 48 h) were embedded in paraffin and sliced on a microtome (4 µm, Leica Biosystems GmbH, Germany). The sections were stained using a BOND-III automated immunostaining system (Leica Biosystems GmbH, Germany). The coronal brain sections (at approximately − 1.6 to − 2.1 mm relative to bregma, 2 mice per line) were stained for Aβ (anti-human Aβ clone 4G8; 1:4000, BioLegend™); TAU (anti-human TAU clone TAU-5, 1:100, Calbiochem); AT8 (anti-human PHF-TAU clone AT8, 1:100, Thermo Scientific); AT180 (anti-human PHF-TAU clone AT180, 1:100, Thermo Scientific); microglia (IBA1, 1:1000, Wako, 019-19741); and astrocytes (GFAP, 1:1000, DAKO, Z033401) as previously described by us [21–27]. The Campbell–Switzer (CS) stains were performed as described in Additional file 2: Method S1. After staining, tissue sections were digitized at 230 nm resolution using a Pannoramic slide scanner (3DHistotech, Hungary) and were subject to specialist evaluation by a neuropathologist.

#### Immunofluorescent staining of frozen brain tissue

Mice were killed at a designated time (transcardially punctured and saline-perfused) and their brains rapidly excised. The two hemispheres were separated and snap-frozen in liquid nitrogen. 10-μm coronal brain sections were prepared using a cryostat and then stained. Slices were fixed in 4% formaldehyde in PBS and then incubated with a blocking solution containing 1% BSA, 8% horse serum and 0.3% triton (Sigma-Aldrich, Israel) in PBS for 20 min at room temperature. Subsequently, the sections were incubated with primary antibodies anti-Tau pSer202 (#28017, 1:500, AnaSpec Inc.), anti-GFAP (G9269, 1:250, Sigma-Aldrich), anti-Abeta (clone 6E10, SIG-39300, 1:750, SIGNET), anti-cluster of differentiation molecule 11B (CD11b) (ab133357, 1:50, Abcam) and OC antibody [28, 29], which was received from Prof. Rakez Kayed’s laboratory, in a blocking solution for 1 h at room temperature. Following three washes with PBS–Tween, sections were incubated with Alexa-fluor 488-conjugated goat anti-rabbit (A-11008), 1:250, Invitrogen) or with Alexa-fluor 594 conjugated goat anti-mouse (A-11005), 1:250, Invitrogen) IgG secondary antibody, for 30 min at room temperature, and then developed as previously reported [20].

#### Congo red staining

In order to further characterize Aβ depositions, brain slices were also stained using the Congo red method, which is a chemical stain for β-sheeted protein structures. Slices were fixed for 1 min in 70% ethanol and rinsed for 2 min in water (ddH_2_O). Then, they were put for 10 min in Congo red solution (0.15 g NaCl, 0.15 g Congo Red [Sigma-Aldrich], 0.5 mL 1% NaOH) in 49.5 mL 80% ethanol.

### Image analysis

Image analysis was performed using Image-Pro software (Version 10.0.4, Media Cybernetics, USA) by an investigator different from the one who conducted the staining and imaging. The calibration was performed based on the scale bar presented in the images. This saved calibration was then applied to each image according to their respective scales before any further processing. To identify GFAP-stained astrocytes within the images, a separate investigator, blinded to the mouse genotype, selected astrocytes for subsequent analysis. The selected astrocytes were delineated as regions of interest (ROI). An optimal threshold was chosen to allow for astrocyte-specific segmentation, and this threshold was consistently applied to all subsequent analyzed images. The software measured the “area” of each segmented astrocyte. For CS-stained plaques, segmentation was performed immediately after calibration for all cortical images. The segments occupying an area below 60 µm^2^ were excluded from the analysis. The measurement settings were configured to return the “area” of each segmented plaque.

### Western blot analysis

Brain tissues from WT, TAU, 5xFAD and 5xFAD TAU mice were cut using a cryostat at 60 μm thickness of the left hemisphere containing the hippocampus; (from − 1.34 mm to − 2.3 mm to bregma). Tissues were homogenized for 20 min in an ice-cold homogenization buffer [37.5 mM NaCl, 50 mM Tris buffer pH 7.6, 20 mM MgCl_2_, 0.5% NP40, 1 mM DTT, 1 mM PMSF, 0.1 mM vanadate (Sigma-Aldrich), 1:100 protease inhibitor cocktail (MBS53913110VL, Mercury and 1:100 phosphatase inhibitor cocktail 1 (P2850; Sigma-Aldrich) and 1:100 phosphatase inhibitor cocktail 2 (P5726; Sigma-Aldrich), centrifuged in 4 °C at 12,000 rpm for 20 min and then supernatants were collected and protein concentration was determined according to Bradford using BSA as a standard as previously described. Blots were prepared as described by Harlow et al. [30] using 12% polyacrylamide gels and then transferred to a polyvinylidene disulfide (PVDF) membrane for 2 h at 260 mA. After a one-hour preincubation with blocking solution containing 3% BSA, blots where incubated overnight with primary antibodies rabbit anti-Glutamine synthetase (G2781; 1:5000, Sigma-Aldrich), rabbit anti-Glial fibrillary acidic protein (GFAP) (G9269, 1:1000, Sigma), rabbit anti-TAU pSer202 (#28017, 1:1000, AnaSpec, Inc.), rabbit anti-VEGF (ab46154, 1:800, Abcam), GSK-3α/β and β-actin were from Santa-Cruz biotechnology (SC-7291, SC-1615). β-catenin antibody was from Transduction Laboratories (#610154) and normalized either to mouse anti-GAPDH (MAB374, 1:10,000, Millipore) or actin at 4 °C. Following three washes with PBS–Tween, sections were incubated with the appropriate secondary antibody goat anti-rabbit/mouse, infra-red (IR) dye conjugated (1:10,000, LI-COR Biosciences GmbH, Germany) for 1 h at room temperature. Blots were scanned using the LI-COR Odyssey imaging system. An analysis of band intensities was done using the Odyssey® software (LI-COR Biosciences GmbH, Germany).

### Aβ quantification

Amyloid-β load was assessed both as total Aβ by using ELISA and by immunohistochemistry approaches. To quantify total Aβ42 by ELISA, the right hemisphere of each mouse in each treatment group was homogenized with PBS containing protease inhibitor and centrifuged at 100,000×*g* for 30 min. The supernatant-containing soluble Aβ was stored at − 70 °C. The pellet containing insoluble Aβ was extracted in 5 M guanidinium-hydrochloride (pH 8) for 3 h at room temperature. Dilutions were used to measure levels of Aβ1-42 by sandwich ELISA (EZBRAIN42, Merck). To quantify Aβ immunohistochemistry, we studied 2 consecutive sections per mice and up to 6 mice per group. Well-defined hippocampal regions (Bregma − 1.94 mm) were selected for quantification of the amount of Aβ and Aβ plaques using antibody or Congo red stains. Slides were observed using a light microscope (Nikon Eclipse 80i, Japan) and fluorescent images were taken using a microscope camera (Micropublisher 6, Teledyne Photometrics, USA). The quantification was done in a blinded fashion to the person who stained the sections using NIS imaging software analysis (Nikon, Japan). Aβ was quantified according to the stained area that was determined by the intensity threshold.

### Primary astrocyte culture

Primary culture was prepared from brains of 1–3-day-old WT vs. TAU pups. In order to determine the genotypes of the newborn mice, we performed the screening procedure as described in Additional file 1: Fig. S1. Cells were grown for 7 days in Dulbecco’s-modified Eagle’s medium (DMEM) supplemented with 10% FCS, 4 mM l-glutamine, 100 units/mL penicillin, 0.1 mg/mL streptomycin (Biological Industries, Beit-Haemek, Israel) and 1% sodium pyruvate (Sigma-Aldrich), and were maintained at 37 °C, 5% CO_2_ and 95% relative humidity. The cells were homogenized and protein expression was assessed.

### Adult microglia isolation

Adult microglia were isolated from 3-month-old TAU or WT mice using the CD11b magnetic beads separation kit (#130-049-601, Miltenyi Biotec) and were analyzed for mRNA expression as previously described [31].

### Adult astrocyte isolation

Adult astrocytes were isolated from 4-month-old mice and processed as previously reported [31].

### RT-PCR

Total RNA was extracted from cells using the MasterPure™ RNA Purification Kit (Epicentre Biotechnologies, USA). SYBR Green real-time PCR primers were purchased from Agentek (Israel). RT-PCR was performed with primers specific for glial fibrillary acidic protein (GFAP), S100β, vascular endothelial growth factor (VEGF), transforming growth factor beta 1 (TGF-β1), tumor necrosis factor (TNFα), interleukin 10 (IL-10), cluster of differentiation molecule 11B (CD11B), scavenger receptor A (SRA), using an PRISM 7300 thermal cycler (Applied Biosystems) as previously described [31].

### Statistical analysis

Results were analyzed using Prism (v9, GraphPad Software Ltd., USA), version 12 (StatSoft, Inc. Tulsa, OK, USA), by either a two-tailed Student’s *t*-test when two groups were compared or a two-way ANOVA (followed by Bonferroni post hoc test) when three or more groups were analyzed. *p*-values less than 0.05 were considered statistically significant.

## Results

### Assessment of the memory impairment of 5xFAD TAU mice

In order to assess the effect of expression of TAU under the endogenous promoter on disease progression in *APP* transgenic mice, we crossbred 5xFAD mice with *MAPT* mice (TAU) [18] to obtain 5xFAD TAU double transgenic mice. Mice were maintained in a 5xFAD hemizygote (5xFAD^+/−^) and *MAPT* hemizygote breeding scheme (*MAPT*^+/−^).

**Fig. 1:**
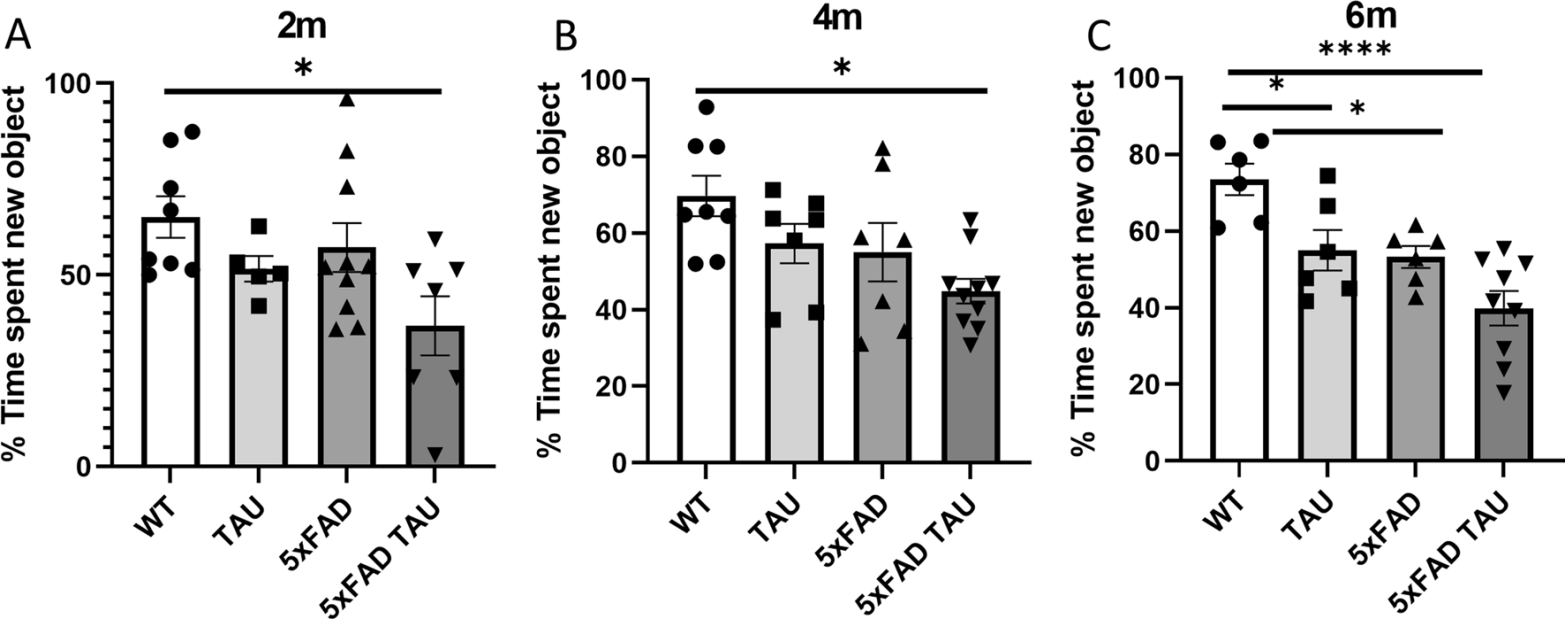
5xFAD TAU mice show early memory impairment as compared to 5xFAD mice and TAU mice. Novel object recognition task was conducted with WT, TAU, 5xFAD and 5xFAD TAU mice at **A** 2, **B** 4, and **C** 6 months of age (*n* = 7–10 mice per group). Statistical analysis was performed using one-way ANOVA, results are presented in mean ± SEM, *p < 0.05

Previous publications have suggested that 5xFAD mice start to develop a behavioral phenotype starting at 4 months of age [19] and the TAU mice at 6 months of age [18]. We aimed to assess whether 5xFAD TAU transgenic mice will exacerbate memory impairment using a novel object recognition test at different ages: 2, 4, and 6 months, respectively, as compared to WT, TAU and 5xFAD mice. Our results (Fig. 1) show a significant reduction at 2 months of age in the exploration of new objects that refers to cognitive impairment only in the 5xFAD TAU mice vs. WT (*p = 0.0217). A significant reduction in memory behavior only in 5xFAD TAU was also observed at 4 months of age (*p = 0.0068). At the age of 6 months, all transgenic mice (5xFAD, TAU, and 5xFAD TAU) showed memory impairment as compared to WT mice (5xFAD TAU mice: *p* < 0.0001; TAU mice: *p* = 0.0492; 5xFAD mice; *p* = 0.028).

### Increase in TAU pathology in 5xFAD TAU mice

**Fig. 2:**
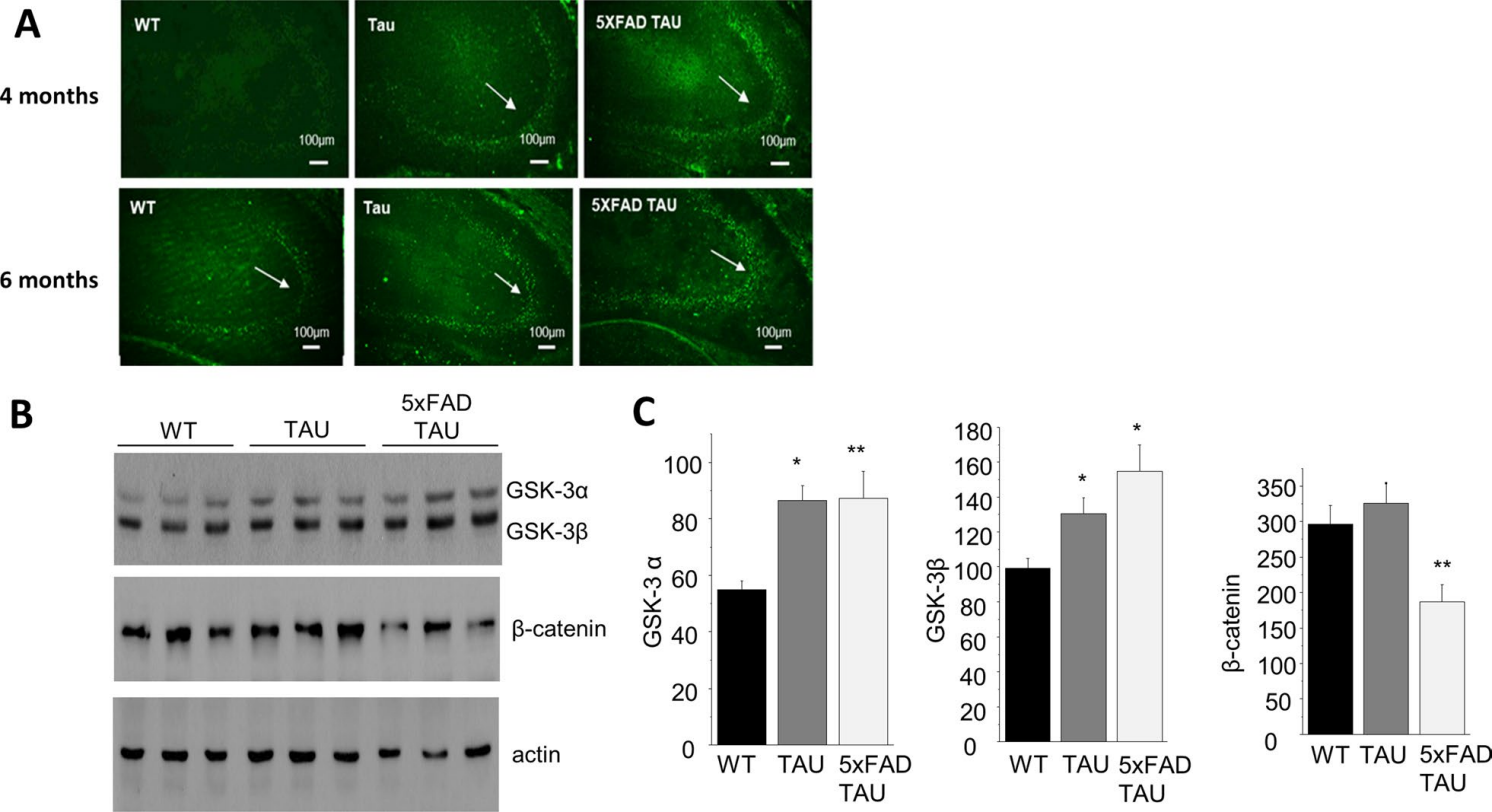
Increase in hyperphosphorylated τ immunoreactivity in hippocampus of 4 and 6-month-old 5xFAD TAU mice vs. controls. **A** Hippocampal sections from WT, TAU and 5xFAD TAU 4- and 6-month-old mice were stained using TAU (pS202) antibody. Hippocampus CA3 region is presented (*n* = 3). Scale bar = 100 µm. **B**, **C** WB analysis of GSK-3 levels in TAU and 5xFAD TAU mice as compared to WT mice, showed elevated expression levels of GSK-3 isozymes, GSK-3α and GSK-3β, respectively. Interestingly, only the 5xFAD TAU mice exhibit significant reduction in β-catenin levels as compared to WT (− 63%, *p* < 0.01), a possible outcome of the increased GSK-3 levels, which targets β-catenin to proteasomal degradation

We further assessed the levels of TAU hyperphosphorylation in the hippocampus of the transgenic mice, using the Tau (pS202) antibody. We discovered (Fig. 2A) that already at 4 months of age, we can identify positive staining in the CA3 of both TAU and 5xFAD TAU mice with increased intensity in the 5xFAD TAU mice. Our results are in line with a previous report that shows positive hyperphosphorylated of TAU in 5xFAD mice [32]. TAU-5 staining (Additional file 1: Fig. S2) shows similar expression levels of total τ in all mouse strains used in this study. This confirms the physiological expression levels of total τ also in the *MAPT* transgenic mice. Of note, AT8 and AT10 antibodies using FFPE sections did not detect any NFTs at this age (6 months). GSK-3β has previously been linked to the phosphorylation process of τ [7]. In order to verify that the immunostaining reflects increased τ phosphorylation, we assessed the changes in GSK isoenzymes, GSK-3α and β, using Western blot and activity in TAU and 5xFAD TAU vs. WT mice (Fig. 2B, C). We found that both TAU and 5xFAD TAU mice had increased brain levels of GSK-3β as compared to WT mice, as demonstrated in the elevated expression levels of GSK-3 isozymes, GSK-3α and GSK-3β. β-Catenin is phosphorylated by GSK-3β resulting in its nuclear translocation and proteasomal degradation [33]. We discovered that only the 5xFAD TAU mice exhibit a significant reduction in β-catenin levels as compared to WT (− 63%, *p* < 0.01), a possible outcome of the increased GSK-3β levels.

### Increase in brain pathology in 5xFAD TAU mice

**Fig. 3:**
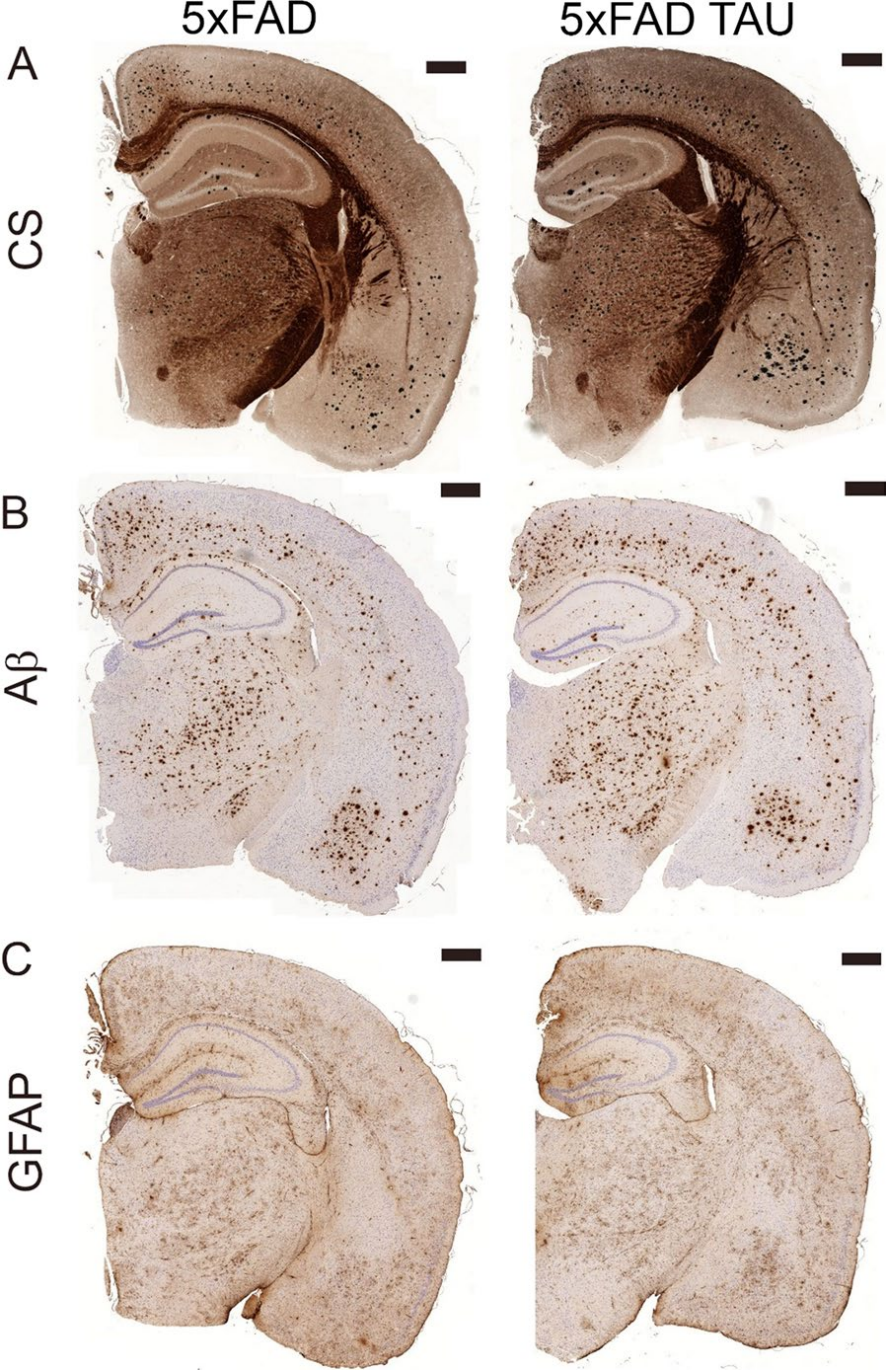
Hemispheric overview of morphological analysis of 5xFAD and 5xFAD TAU mice (− 1.6 to − 2.0 mm from bregma) using silver and immunohistochemical stains. **A** Campbell–Switzer stain showing neuritic pathology and amyloid deposits; **B** Aβ stain showing plaque pathology; **C** GFAP stain showing astrocyte activation and morphology. Scale bars 500 µm

**Fig. 4:**
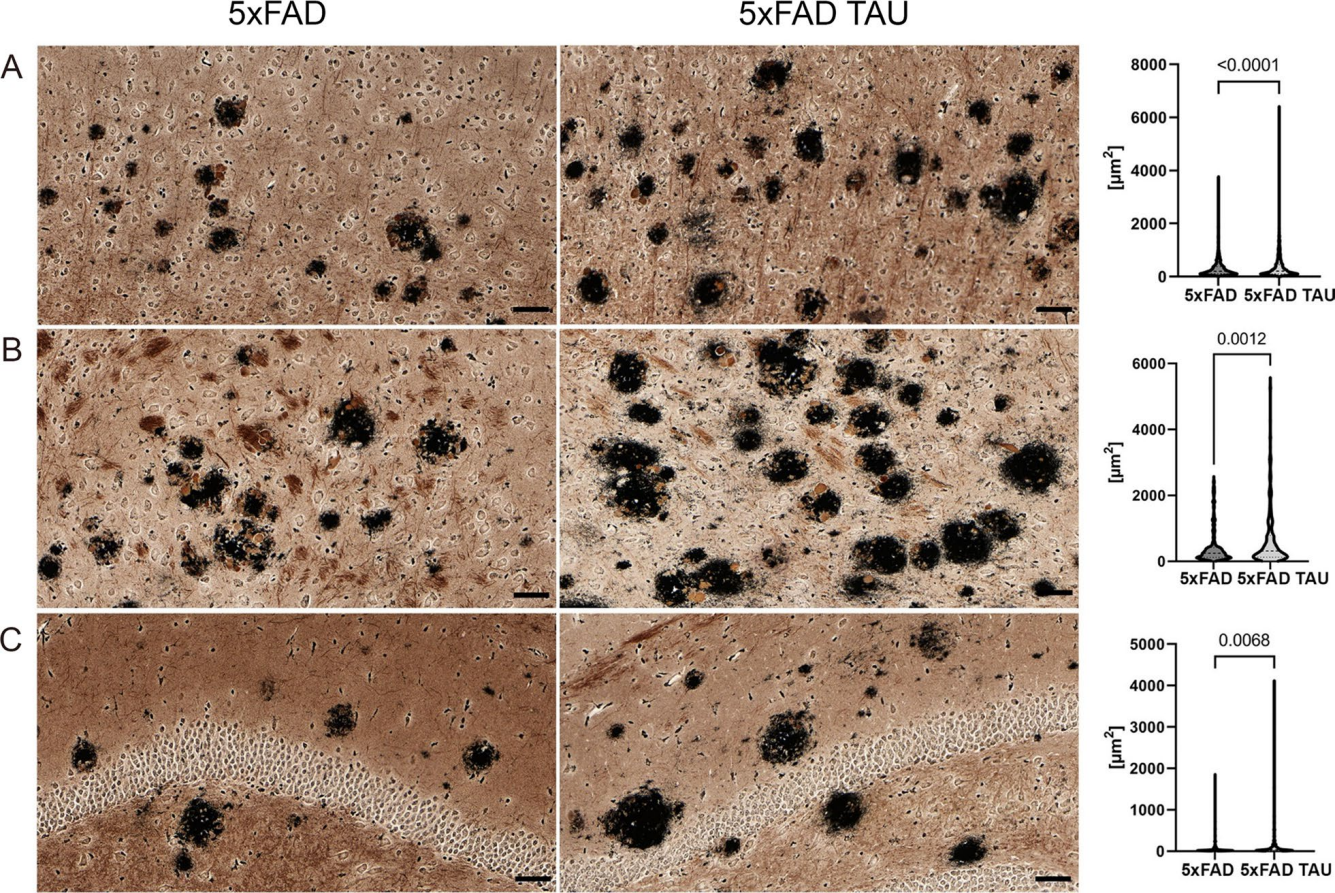
Representation of neuritic pathology using Campbell–Switzer silver stain. **A** Cortex regions, **B** amygdala, and **C** hippocampus. 5xFAD TAU mice show larger and more intense neuritic halos (dark stain) around amyloid plaques. Quantification of average size in µm^2^ of each staining per area (for each mouse model the following CS plaques bigger than 60 µm^2^ were selected: cortex, *n* = 1000–2000; amygdala, *n* = 200–300; and hippocampus, *n* = 100–130) was calculated using Image-Pro software (Version 10.0.4, Media Cybernetics). Scale bars 40 µm

**Fig. 5:**
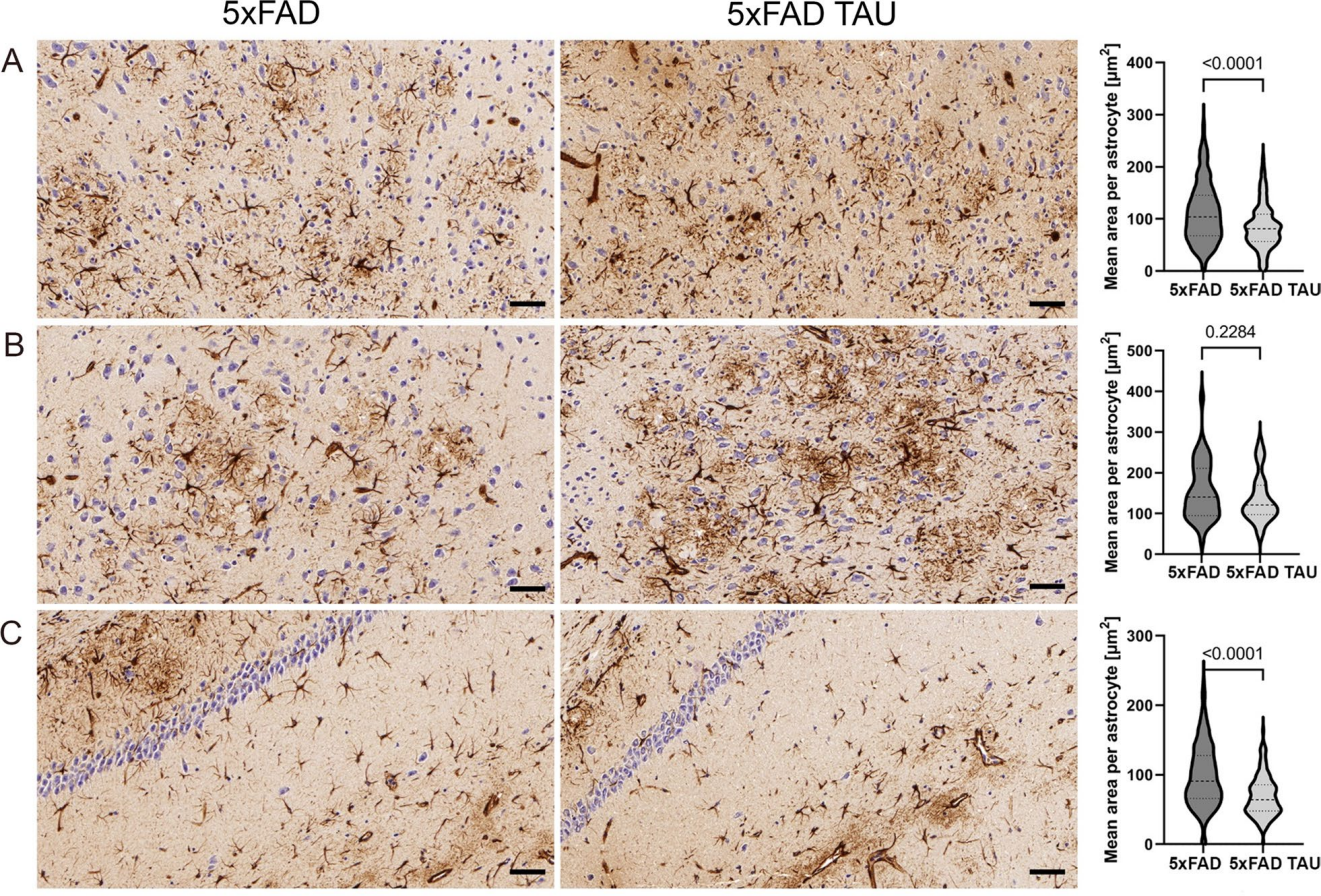
Representation of astrocyte morphology using anti-GFAP stain. **A** Cortex regions, **B** amygdala, and **C** hippocampus. 5xFAD TAU mice show impaired and reduce area size of astrocytes size in brain region of 5xFAD TAU mice vs 5xFAD mice around amyloid plaques. Quantification of average size in µm^2^ of each staining per area was calculated by Image-Pro software (version 10.0.4, Media Cybernetics). For each mouse model the following numbers of astrocytes were selected: cortex *n* = 100–120, amygdala *n* = 6–8; hippocampus *n* = 140–160. Scale bars 40 µm

**Fig. 6:**
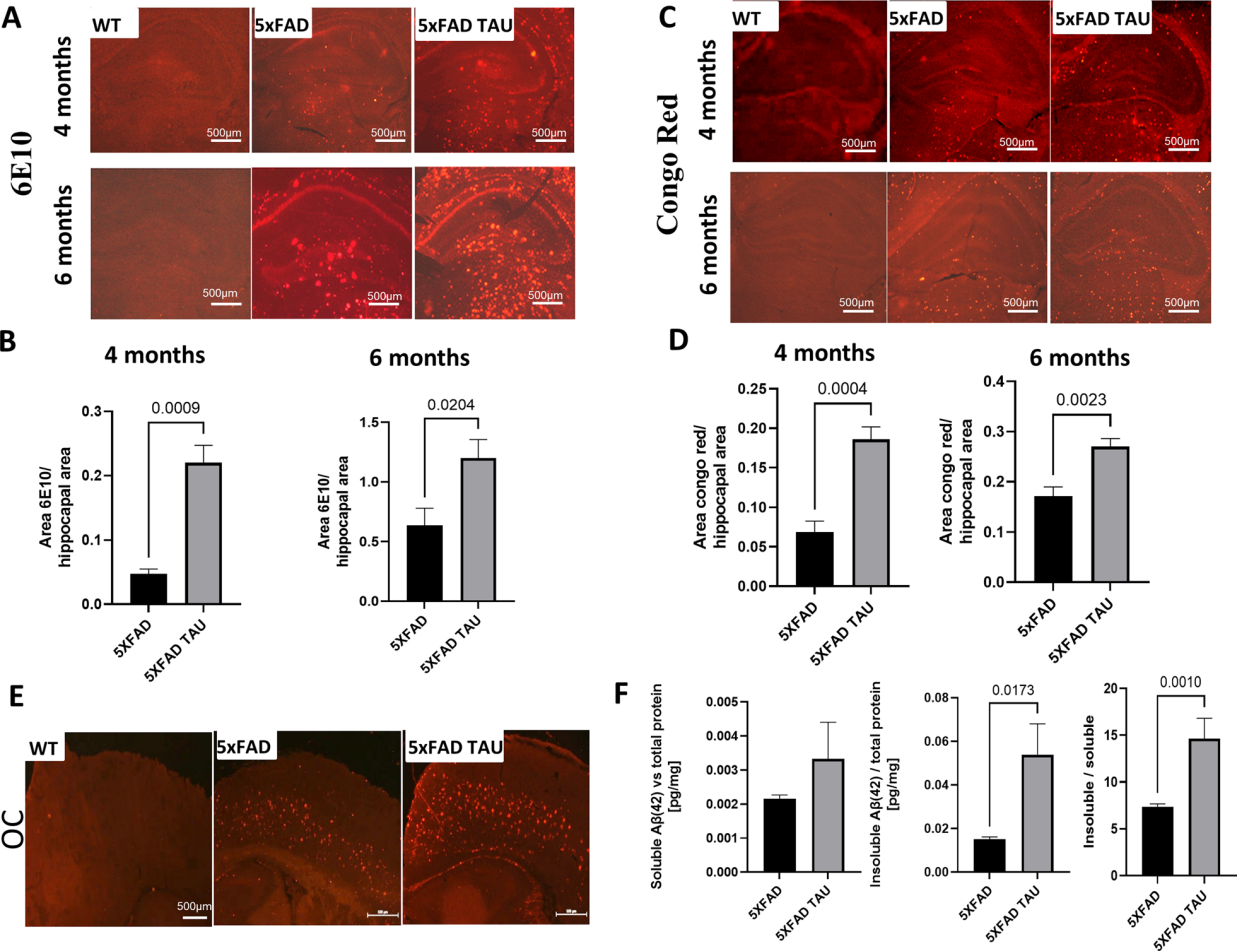
Increase is Aβ burden in brains of 4 and 6-month-old 5xFAD TAU vs. 5xFAD mice. Hippocampal sections from WT, 5xFAD and 5xFAD TAU mice were stained using 6E10 antibody. **A** Representative images of 4- and 6-month-old mice, Scale bar = 100 µm and **B** quantification of 4- and 6-month-old transgenic mice. Statistical analysis was performed as area of 6E10 staining vs hippocampal region, results are presented in mean ± SEM, *n* = 4–6; **C**, **D** Congophilic plaque histological analysis of brains of 4- and 6-month-old transgenic mice. Hippocampal sections from mice were stained with Congo red (statistical analysis was performed using one-way ANOVA, results are presented in mean ± SEM n = 6, Scale bar = 100 µm). **E** Staining with OC antibody, specifically recognizes fibrils amyloid in the cortex of TAU, 5xFAD and 5xFAD TAU mice Scale bar = 100 µm, and **F** ELISA assessment of soluble Aβ42 and insoluble Aβ42 vs total brain and the Aβ ratio between insoluble to soluble Aβ42 in 5xFAD TAU vs 5xFAD mice (*n* = 6–7)

We further evaluated changes in brain pathology attributed to the expression of mutated TAU. Of note, one of the unique features of our model is the expression of the mutated TAU under the *natural* mouse *Tau*-promoter. We specifically focused on three different markers assessing (i) development of neuritic plaque pathology [Campbell–Switzer stain, CS); (ii) Aβ accumulation in general (Aβ stain, clone 4G8); and (iii) changes in astrocyte cell body morphology (GFAP)]. Of note, CS is used for routine diagnostic pathology in patients (see Braak and Braak staging [2]) (Figs. 3, 4, and 5). As presented in Fig. 4, we detected an increase in the CS staining area that has previously been linked to neuritic plaque pathology in human [2], in 5xFAD TAU mice vs. 5xFAD mice at 6 months. We saw a specific increase of neuritic plaques in the cortex (Figs. 3A and 4A), amygdala (Figs. 3A and 4B) and the hippocampus (Figs. 3A and 4C). We discovered that the average size of the CS staining area was significantly increased in 5xFAD TAU vs 5xFAD mice in all the measured areas (Fig. 4). These results suggest increased neurofibrillary changes in the dendrites/axon that are in close vicinity to Aβ deposits as has previously been suggested [23, 24, 27, 34]. This phenomenon was linked to the general increase in Aβ depositions (plaque number and size) (Figs. 3 and 6). In parallel with the increase of neuritic pathology, we discovered pathological changes in astrocytes cell body morphology (Fig. 5) as was assessed by a significant reduction in the areas covered by individual astrocytes from cortex, amygdala and hippocampus of 5xFAD TAU mice vs 5xFAD mice.

### Increase in Aβ pathology in 5xFAD TAU mice

We next aimed to evaluate the levels of Aβ pathology. Aβ plaques are formed from β-amyloid fibrils, which are characterized by a high content of β-sheet structure. For the evaluation of the Aβ plaques of 4- and 6-month-old brains from WT, 5xFAD and 5xFAD TAU mice, we performed two types of staining: (i) specific Aβ immunohistochemical analysis using an Aβ antibody stain (Figs. 3 and 6A, B); (ii) to determine general amyloidosis seen in AD, we used Congo red staining (Fig. 6C, D) which is a lipophilic dye that binds to the β-sheet structure that composes the dense regions of Aβ plaques. Interestingly, we observed starting at 4 months of age a significant increase in both insoluble Aβ42 deposition (5xFAD TAU vs. 5xFAD mice, *p* = 0.0009) and congophilic β-amyloid (5xFAD TAU vs. 5xFAD mice, *p* = 0.0204) (Fig. 6C, D). The same phenomenon was found at 6 months of age (insoluble Aβ42 deposition 5xFAD TAU vs. 5xFAD mice, *p* = 0.0204) and congophilic β-amyloid (5xFAD TAU vs. 5xFAD mice, *p* = 0.0023) (Fig. 6C, D).

The OC is a polyclonal rabbit antibody that specifically recognizes fibrils, but not random coil monomer or prefibrillar oligomers [35]. The fibril-specific antibodies stain all types of amyloid deposits in a human AD brain. We investigated the distribution of OC staining in our model mice. We found (Fig. 8E) an increase in positive staining in the cortex of 5xFAD TAU mice vs. 5xFAD mice. We further examined changes within brain soluble and insoluble Aβ as compared to total protein in 5xFAD TAU mice vs 5xFAD mice using ELISA and discovered (Fig. 6F) a trend of increased soluble Aβ and significant increase in insoluble Aβ vs soluble Aβ ratio (*p* = 0.0173 and *p* = 0.001, respectively).

### Changes in the morphological appearance of astrocytes in 5xFAD TAU mice

**Fig. 7:**
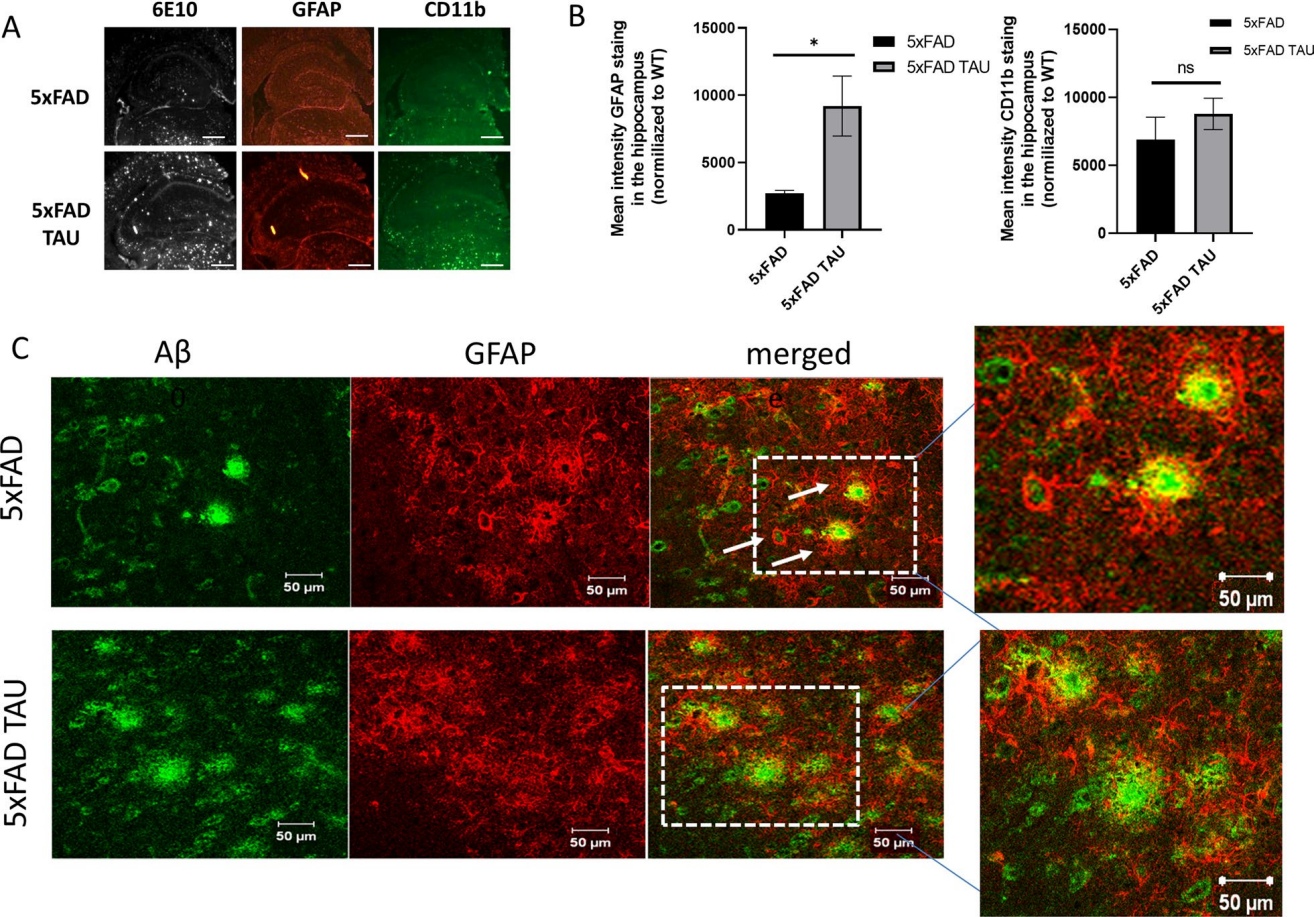
Dystrophic appearance of astrocytes in 5xFAD TAU mouse brains. **A** Immunofluorescent staining of Aβ (6E10), astrocytes (GFAP) and microglia (CD11b) in 5xFAD and 5xFAD TAU mice, Scale bar = 500 µm. **B** Quantification of mean intensity of GFAP and CD11b-positive staining within the hippocampus of 5xFAD vs. 5xFAD TAU mice normalized to WT. **C** Co-staining of GFAP-Aβ in the brain of 5xFAD vs. 5xFAD TAU mice show dystrophic appearance of astrocytes in 5xFAD TAU mice around Aβ plaques. Scale bar = 50 µm

**Fig. 8:**
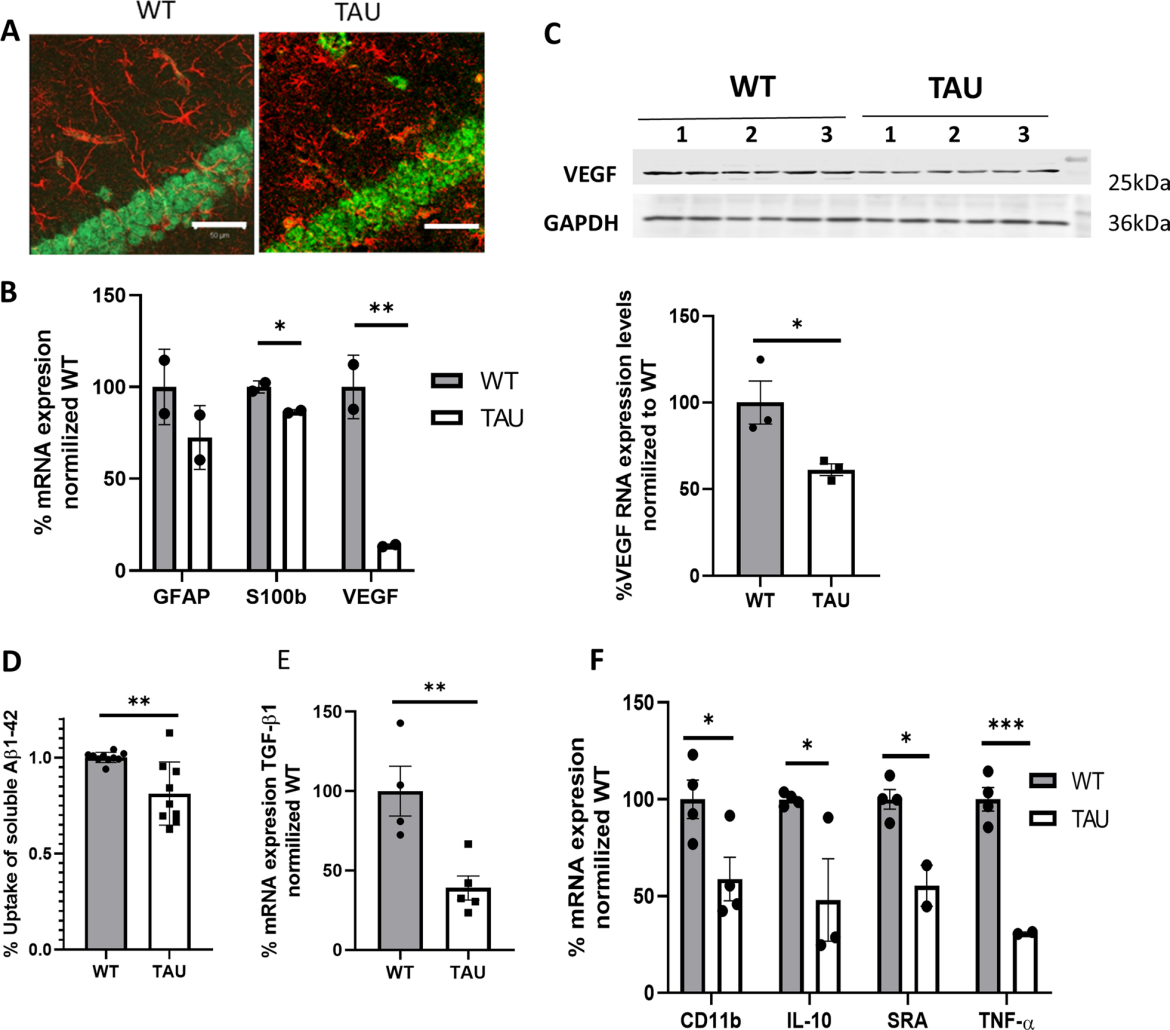
Expression of mutated TAU in astrocytes impaired their morphology and activity. Astrocytes were isolated from mice expressing mutated TAU and compared to WT C57BL6/J mice. Scale bar 50 µm. **A** Co-staining with GFAP (red) and NeuN (green) of brain from 3-month-old WT and TAU mice; **B** RT-PCR analysis of GFAP, S100b and VEGF from primary astrocytes from WT and TAU mice (*n* = 2 repeats per group, each repeat is based on 2 mouse brains); **C** VEGF protein levels in Tau mutated primary astrocytes compared to WT astrocytes. Protein expression was determined and was taken from 1- to 3-day-old WT vs. TAU newborn mice (*n* = 3 repeats per group, each repeat is based on 2 mouse brains. Representative bands and Protein quantification. Statistical analysis was performed using Student’s *t*-test, results are presented in mean (% fold increase of control); **D** FACS analysis of uptake of fluorescent Aβ42 by adult astrocyte from WT and TAU mice (*n* = 8–9); **E** mRNA analysis of TGF-β1 expression by adult astrocyte from WT and TAU mice (*n* = 4–5); and **F** mRNA expression levels analysis from adult microglia isolated by CD11b magnetic beads from 3-month-old WT and TAU mice (*n* = 3–4, each repetition is based on 2 mice; *p < 0.05; **p < 0.01, ***p = 0.005)

We further investigated the activation of glia cells in 5xFAD TAU mice. We analyzed the mean intensity of microglia (CD11b) and astrocytes (GFAP) at 6 months of age (Fig. 7). We found that while there is not a significant difference between CD11b-positive staining in the hippocampus of 5xFAD TAU vs. 5xFAD mice, there was an elevation in GFAP positivity in 5xFAD TAU vs. 5xFAD mice (*p* = 0.0491, Fig. 7A, B). Nevertheless, co-staining of Aβ and GFAP revealed a dystrophic appearance of the astrocytes (Fig. 7C) surrounding Aβ plaques. While the astrocytes in 5xFAD seem to tightly surround the Aβ plaques, those in 5xFAD TAU mice seem to have an impairment to engulf the plaques that resulted in an amorphic appearance. We further assessed the role of mutations in TAU on astrocytes activity. It has previously been reported that C57BL6/J mice express τ in astrocytes [13]. We stained 3-month-old TAU mice vs. WT mice, both with C57BL6/J background, for GFAP and found a dystrophic appearance of astrocytes (Fig. 8A). To assess whether a mutation in TAU would affect the maturation of astrocytes at an early stage, we isolated primary astrocytes for cell culture and assessed gene expression (Fig. 8B). We discovered a significant reduction in S100b and VEGF expression. VEGF is a known vascular growth factor produced by activated astrocytes. VEGF gene expression was decreased by 27% (*p* = 0.011) and by 46.65% in the protein levels of VEGF in mutated TAU primary astrocytes vs. WT astrocytes (*p* < 0.05) (Fig. 8B, C). To assess the ability of adult astrocytes to uptake Aβ, we isolated astrocytes from 5 and 5xFAD TAU mice as previously described. We discovered a significant reduction within their ability to uptake soluble Aβ42 as assessed by FACS (Fig. 8D). Interestingly, adult astrocytes also showed a significant reduction in the expression of Tgfβ1, an important trophic factor that was reported to be crucial for microglia maturation. Indeed, as shown in Fig. 8E, adult microglia isolated from TAU mice showed a marked reduction in gene expression of activator markers (Fig. 8F). Adult microglia cells were separated by magnetic beads targeted for CD11b from WT and TAU brains. Our results demonstrate a decrease in microglia activation markers such as CD11b (− 24.3%, *p* = 0.0083), a decrease in TNF-α (− 38.2%, *p* = 0.0011). IL-10 is an anti-inflammatory marker and it is secreted in the protective phenotype of microglia. We report a decreased of − 48.3% (*p* = 0.0002) as compared to WT cells (Fig. 8F). Here, we found that hyperphosphorylation of mutated TAU in neurons, astrocytes and microglia, demonstrates its effect on the gene expression profile and morphology of astrocytes and of microglia cell activity and therefore suggests a non-cell autonomous role of phosphorylation TAU in brain pathology.

## Discussion

In this work, we established and characterized a new combined *APP*, *PS1* and *MAPT* transgenic AD mouse model (5xFAD TAU). This new mouse model presents many features of the disease pathology, while expressing human TAU under the endogenous *Tau*-promoter of the mice. Therefore, it is a more suitable model for AD than other existing animal models. We found that in this model the activity of astrocytes is impaired, and this impairment is attributed to the presence of hyperphosphorylated TAU in the astrocytes.

The amyloid-cascade hypothesis stipulates that Aβ is the trigger of all cases of AD and that the τ pathology and other degenerative changes are a downstream consequence of the initial Aβ pathology [36]. According to this hypothesis, in all AD mouse models found today, which combine *APP* mutations with *MAPT* mutations, extracellular Aβ depositions precede τ pathology. Furthermore, in these models, extracellular Aβ depositions seem to aggravate τ pathology. A similar pathological progression was demonstrated in the 3xTg-AD mouse model generated by Oddo et al*.* [37]. Further validation for this hypothesis was when Oddo et al*.* [37] showed that the removal of Aβ by immunotherapy leads to the removal of early τ pathology. Furthermore, after intrahippocampal injection of anti-Aβ antibodies, the clearance of Aβ precedes the clearance of the early τ pathology [38]. Nevertheless, the 3xTg-AD mouse model was constructed from a *APP* mouse model that shows pathology at 10 months of age and a *Tau* mouse model that shows pathology at 20 months (amyloid precursor protein (*APP*_swe_), presenilin-1 (PS1_M146V_), and tau_P301L_) [37]. Therefore, this model lacks the ability to address the role of hyperphosphorylation of τ on Aβ levels. Here, the mouse model was constructed form *APP* mice that exhibit Aβ pathology at the age of 4 months and *MAPT* transgenic mice that show τ pathology at the age of 6 months. We found that in our 5xFAD TAU mouse model, tau hyperphosphorylation levels at an age of 6 months were greater than the ones we found in the *TAU* transgenic mice without APP transgene expression [18]. While this finding is consistent with the amyloid hypothesis, we also found that the extracellular Aβ burden was much more severe in our 5xFAD TAU mice than in the 5xFAD mouse model, which suggests a link between τ and Aβ. Our results are in line with reports that τ pathology starts in some AD brain areas independent of Aβ and that later τ and Aβ pathology interact [39]. Furthermore, a previous publication has suggested that a deficiency in τ expression might affect brain amyloid levels [40]. Also, a recent publication suggests that reducing tau pathology with a phos tau peptides vaccine in an amyloid model was associated with a decrease in amyloid pathology, accompanied by a microglial response [41]. Another publication also suggests that interference with the interaction between τ and Aβ might be used as therapeutic application to reduce brain amyloid levels and to improve cognition in 5xFAD mice [42].

We found in 5xFAD TAU mice as compared to 5xFAD mice, an increase in neuritic plaques in the cortex, which suggests increased axonal stress. We propose that our 5xFAD TAU model exhibits neurodegenerative processes that are found in brains from AD patients. With the 5xFAD TAU mice, we found a reduction in exploration behavior at an early age of 2 months. This reduction was not found in the other animals (WT, 5xFAD) at this age. This decrease in 5xFAD TAU mice continued at 4 months and at 6 months of age the reduction further continued. For the first time, it was also apparent at 6 months of age in the TAU mice and the 5xFAD mice.

Of note, TAU mice [18] show at 6 months a reduction in cognitive performance in the eight arm maze. This indicates impairments in the spatial, reference and working memory. In the T-maze, a reduction in working memory was reported at 10 months of age, indicating deficits related with hippocampal dysfunction. As mentioned above, when we tested TAU mice in the novel object recognition test, we found a reduction in memory already at 6 months of age. This indicates dysfunction in spatial and visual memory, related to the hippocampus.

Astrocytes provide essential services for brain homeostasis and neuronal function, including metabolic support for neurons, regulation of local concentration of neurotransmitters and ions, maintaining the integrity of the blood–brain barrier (BBB) and are important in the creation and maintenance of synapses [43]. Thus, any dysfunction or impairment in their activity can result in various different pathologies [43]. Here, we show that that primary astrocyte cultures prepared from mice that expressed human mutated TAU under the *Tau* endogenous promoter have an impairment to express cytoskeleton protein GFAP and neuronal and endothelial cells, important growth factor such as VEGF. Indeed, it has been reported that the expression of mutated TAU under the GFAP promoter in astrocytes resulted in BBB insult and neuronal degeneration [17]. Additionally, VEGF is a neuroprotective growth factor, which is expressed in astrocytes and other cells [44]. We have previously shown reduced expression of VEGF in aged astrocytes that are linked to their impairment to support neuronal growth [45]. Under pathological conditions, it was shown that a reduction in VEGF levels can cause neurodegeneration in part by impairing neural tissue perfusion [46]. A reduction in VEGF has previously been suggested to play a role in AD [47]. Therefore, the reduction we found in this neuroprotective growth factor can also lead to neuronal loss.

Astrocytes are known to be in close association with amyloid plaques [48]. Furthermore, it has previously been shown by Wyss-Coray et al*.* that activated astrocytes are able to accumulate and even degrade fragments of Aβ [49]. Thus, astrocytes that are less activated or suffer activation impairments can perhaps lead to less clearance of Aβ depositions. This can eventually lead to more accumulation of Aβ depositions as seen in our 5xFAD TAU mice. Indeed, in our 5xFAD TAU mice we can see an impairment in the astrocytes ability to engulf the plaques, which results in an increase in brain Aβ load. Of note, it was previously reported that 5xFAD with depletion of TAU show reduction in Aβ load in their brain suggesting a strong link between TAU to Aβ. Here we suggest that the link reflects the role of TAU in maintaining astrocytes activity to facilitate clearance of Aβ.

Here, we demonstrate the role of mutated human TAU expression in astrocytes in the impairment of their morphology and activity. We discovered that expression of TAU pathological changes in astrocytes cell body morphology, and demonstrate its effect of increase amyloid pathology in amyloid load in Fig. 7 and increase in neuritic plaques TAU is expressed in astrocytes as can be seen in the literature both in mice and human [50]. While astrocytes in human brains show an accumulation of hyperphosphorylated TAU in different tauopathies [12], here we suggest that altering astrocyte activity in disease is linked to an impairment of TAU to support cell activity and morphology that may results in exacerbate brain amyloid pathology.

Of note, it has previously been suggested that stress conditions, such as starvation can lead to hyperphosphorylation of TAU in the normal brains of mice [51]. Furthermore, the antibody recognized phosphorylated process that may be linked to stress and to the process affiliated either with the senescence process, which is affiliated with age at the hippocampus [52]. Therefore, we suggest that in brain diseases where there is no direct link to mutation in TAU, such as AD, stress conditions can affect hyperphosphorylation of TAU in astrocytes and alter their activity. Of note, hyperphosphorylation of TAU within astrocytes was also found in Huntington’s disease [53].

Interaction between astrocytes and microglia are essential for maintaining homeostasis and to cope with pathological conditions. An impairment with this interaction might aggravate pathology [54]. Here, we report that astrocytes expressing mutated TAU show impairment in expression of TGF-β1, an important modulator for microglia activity [55]. Additionally, we found that microglia in mice expressing mutated human TAU show an impairment to express factors essential for their activation such as CD11b, and SRA, that limit their ability to clear Aβ [56, 57]. Of note, changes in mRNA expression profiles in microglia has been reported in different brain pathologies, among them amyloid-like pathology [58–63]. Our results suggest that τ may play an essential role of orchestrating not only amyloid deposition and neuronal activity but also glia activity, and impairment in its activity may play a crucial role in AD pathology.

In conclusion, further understanding the crucial role of TAU in orchestrating brain activity in specific astrocytes may suggest a new target for treatments of neurodegenerative diseases such as AD.

## Supplementary Information

**Additional file 1: Figure S1.** Scheme for generating the 5xFAD TAU transgenic mouse model. The heterozygous Tau mice were bred with the heterozygous 5xFAD and yielded 25% WT, 25% *MAPT*, 25% 5xFAD and the new desired 5xFAD TAU mice. PCR products were run in Agarose gel. The arrows show the fragment size of APP, PS1 gene and the *MAPT*gene. **Figure S2.** Total τ expressionin WT, Tau, 5xFAD and 5xFAD TAU mice reveals physiological levels of τ in all models. Note the slight negative stain circular regions in layer 4/5in 5xFAD and 5xFAD TAU mice due to extensive amyloid deposits. **Figure S3.** Pictures of gels from figure 2B. Image processing- The bands shown represent gels from the same experiments. All bands were taken from the same gel. **Figure S4.** Pictures of gels from figure 8C. Image processing. All bands were taken from the same gel.

**Additional file 2. Method S1.** Total Detailed protocol of the Campbell-Switzer Alzheimer silver stain.

## Data Availability

The original data are available from the corresponding authors upon request. Histological panels images are available at 10.17605/OSF.IO/VWQ58. Digital slides (mrxs format) are available upon request to J.P.

## Abbreviations

AD: Alzheimer’s disease
APP: Amyloid-β precursor protein
Aβ: Amyloid-β
CD11b: Cluster of differentiation molecule 11B
CBD: Corticobasal degeneration
FTDP-17: Frontotemporal dementia with Parkinsonism linked to chromosome 17
GFAP: Glial fibrillary acidic protein
GSK3β: Glycogen synthase kinase-3β
IR: Infra-red
*Mapt*: Mouse microtubule-associated protein tau gene
*MAPT*: Human microtubule-associated protein tau gene
NFT: Neurofibrillary tangle
PiD: Pick’s disease
PS-1: Presenilin-1
PSP: Progressive supranuclear palsy
Tau: Mouse τ protein
TAU: Human τ protein
τ: Tau protein in general (mouse or human)
