# Supplementary material for: Physiological expression of mutated TAU impaired astrocyte activity and exacerbates β-amyloid pathology in 5xFAD mice
Source: J Neuroinflammation. 2023 Jul 26;20:174. doi: 10.1186/s12974-023-02823-9 (PMC10369740; doi:10.1186/s12974-023-02823-9)
Supplement: Supplementary file 1 — Additional file 1: Figure S1. Scheme for generating the 5xFAD TAU transgenic mouse model. The heterozygous Tau mice were bred with the heterozygous 5xFAD and yielded 25% WT, 25% MAPT, 25% 5xFAD and the new desired 5xFAD TAU mice. PCR products were run in Agarose gel. The arrows show the fragment size of APP, PS1 gene and the MAPTgene. Figure S2. Total τ expressionin WT, Tau, 5xFAD and 5xFAD TAU mice reveals physiological levels of τ in all models. Note the slight negative stain circular regions in layer 4/5in 5xFAD and 5xFAD TAU mice due to extensive amyloid deposits. Figure S3. Pictures of gels from figure 2B. Image processing- The bands shown represent gels from the same experiments. All bands were taken from the same gel. Figure S4. Pictures of gels from figure 8C. Image processing. All bands were taken from the same gel. [file 12974_2023_2823_MOESM1_ESM.pptx]

## Slide 1
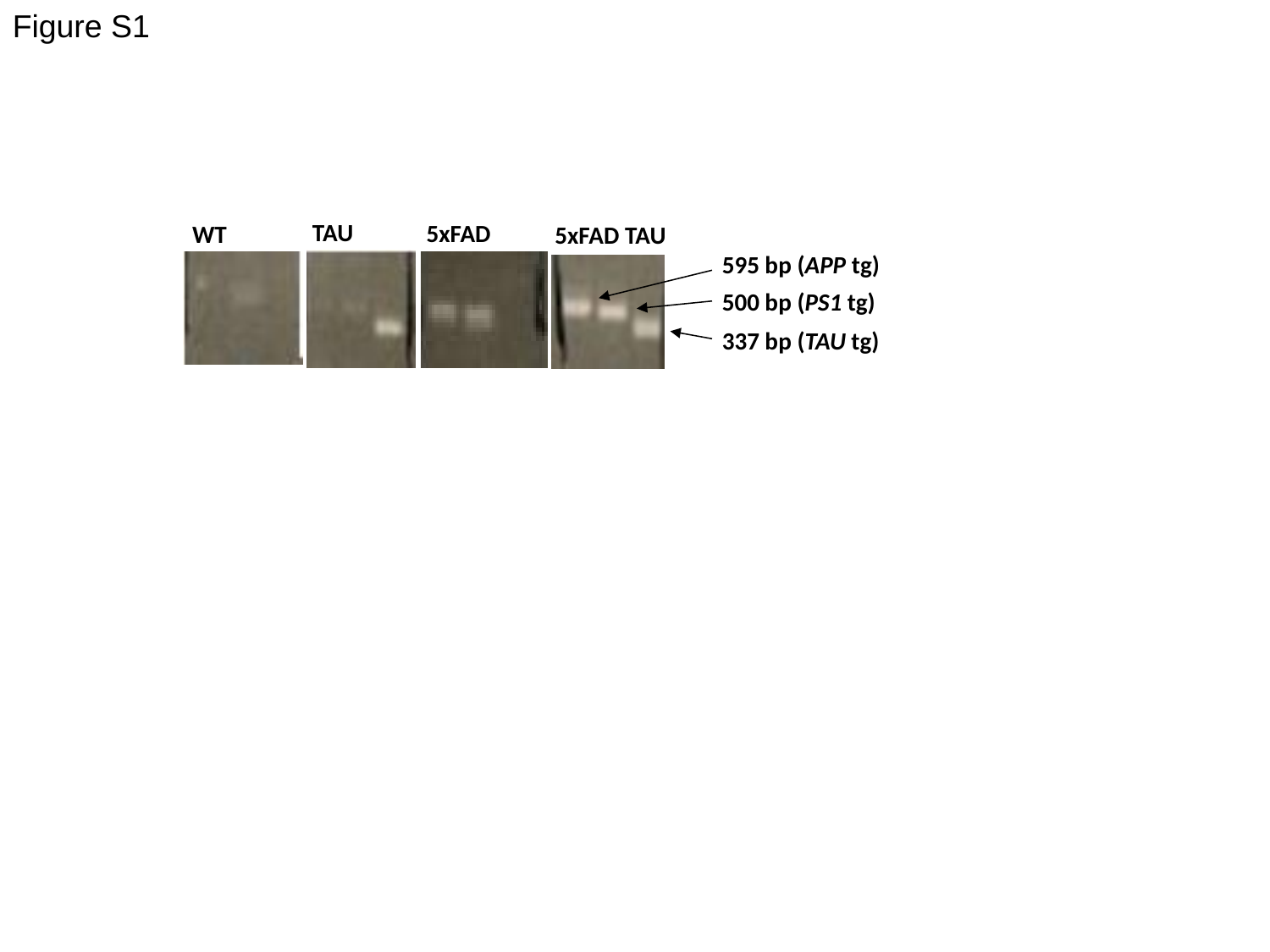

Figure S1
TAU
5xFAD
WT
5xFAD TAU
595 bp (APP tg)
500 bp (PS1 tg)
337 bp (TAU tg)

## Slide 2
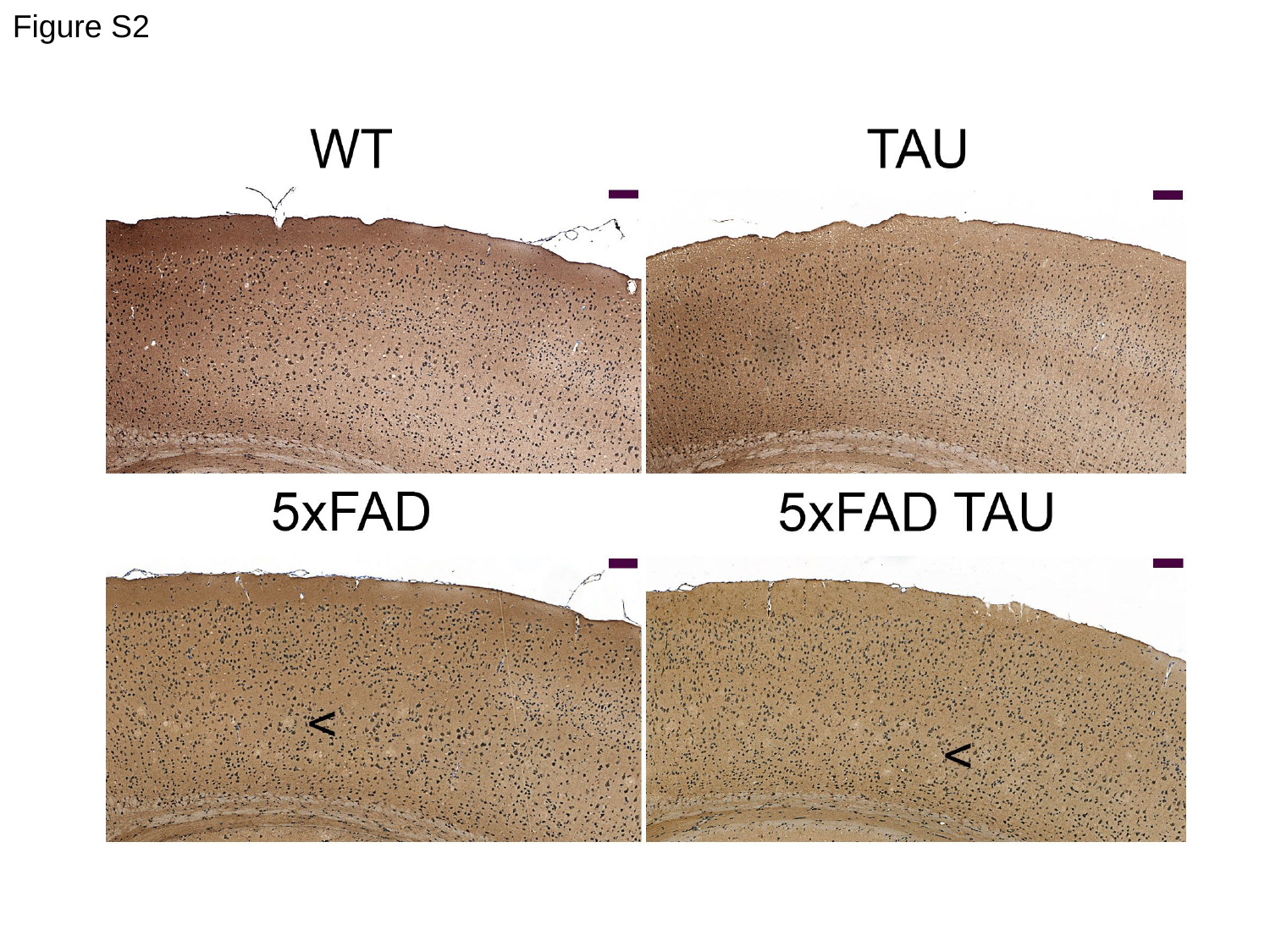

Figure S2

## Slide 3
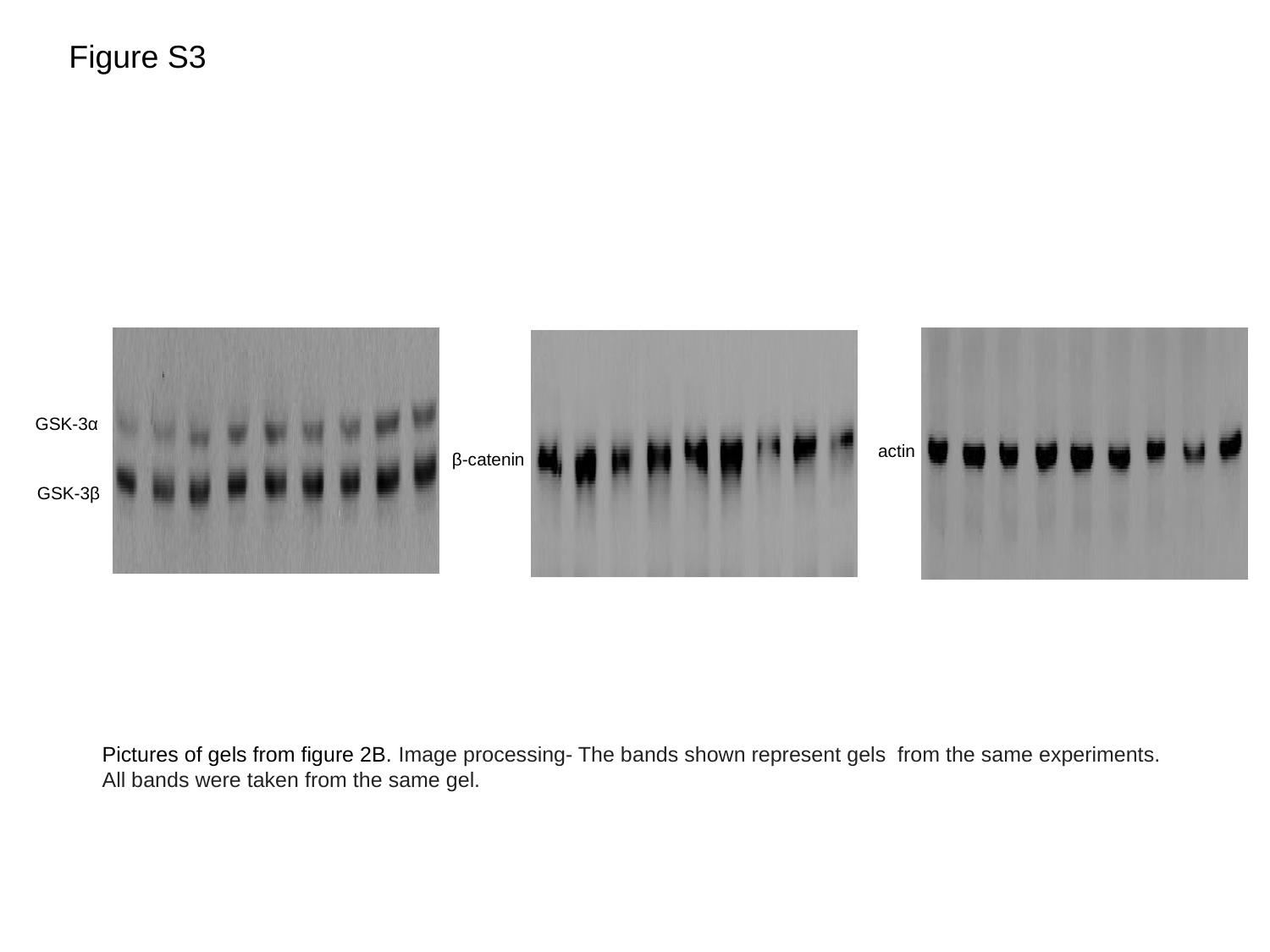

Figure S3
GSK-3α
actin
β-catenin
GSK-3β
Pictures of gels from figure 2B. Image processing- The bands shown represent gels from the same experiments.
All bands were taken from the same gel.

## Slide 4
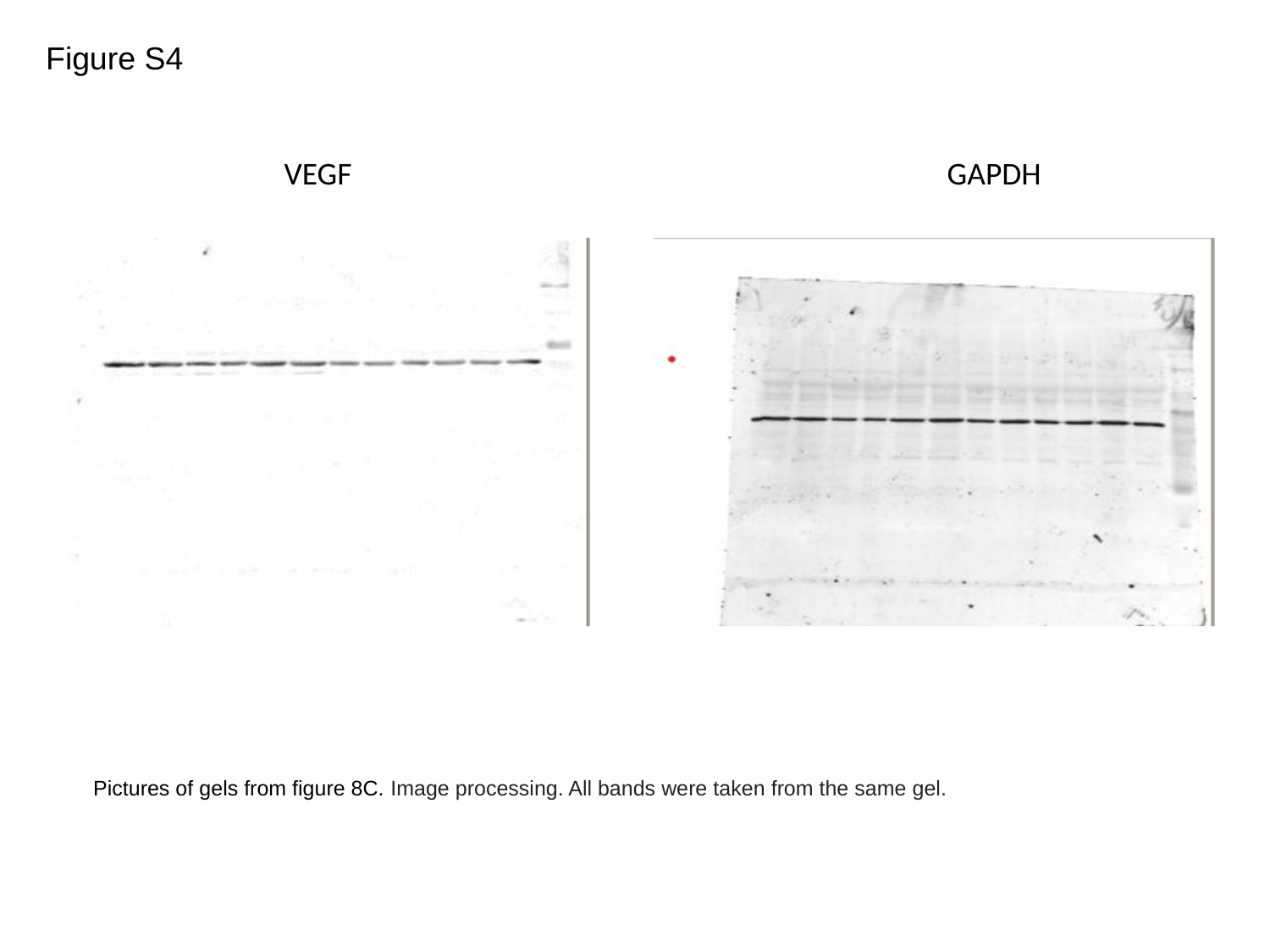

Figure S4
VEGF
GAPDH
Pictures of gels from figure 8C. Image processing. All bands were taken from the same gel.
