# Supplementary material for: Physiological expression of mutated TAU impaired astrocyte activity and exacerbates β-amyloid pathology in 5xFAD mice
Source: J Neuroinflammation. 2023 Jul 26;20:174. doi: 10.1186/s12974-023-02823-9 (PMC10369740; doi:10.1186/s12974-023-02823-9)
Supplement: Supplementary file 2 — Additional file 2. Method S1. Total Detailed protocol of the Campbell-Switzer Alzheimer silver stain. [file 12974_2023_2823_MOESM2_ESM.docx]

**Additional file 2**

**Additional methods**

1. **Campbell-Switzer Alzheimer Silver Staining**

**Protocol:**

1. Prepare Physical Developer Solutions A, B & C
2. Deparaffinize and rehydrate sections and place in dH2O 3 x 10 min each.
3. Prepare SPC Solutions. Stir until needed. Prepare other solutions.
4. Place sections in 2% NH4OH. Stir for 5 min.
5. Place in dH2O 2 x 1 min. each.
6. Place in the SPC solution and gently stir for 40 min.
7. Place in 1% Citric Acid for 3 min.
8. Place in 4.99 pH Acetate Buffer Working Solution until ready for Physical Developer.
9. Prepare fresh Physical Developer ABC solution.
10. Place in Physical Developer ABC solution over a light source. The development time is visually assessed.
11. Stop development by placing in 4.99 pH Acetate Buffer Working Solution briefly.
12. Place in fresh 4.99 pH Acetate Buffer Working Solution. Timing is not critical.
13. Place into fresh dH2O 30 sec.
14. Place into 0.5% Sodium Thiosulfate solution for 45 sec.
15. Place into fresh dH2O 3 x 2 min. each
16. Dehydrate sections.
17. Coverslip.

**Solutions:**

| **1% Silver Nitrate (AgNO3)** |
| --- |
| 100 ml dH2O |
| 1.0 g AgNO3 |
| 100 ml 1% AgNO3 |

| **1% Potassium Carbonate (K2CO3)** |
| --- |
| 100 ml dH2O |
| 1.0 g K2CO3 |
| 100 ml 1% K2CO3 |

| **Silver-Pyridine-Carbonate (SPC)** |  |
| --- | --- |
| 1% AgNO3 | 120 ml |
| Pyridine | 34 ml |
| 1% K2CO3 | 90 ml |
| Total Volume: | 244 ml |

| **2% Ammonium Hydroxide** |
| --- |
| 98 ml dH2O |
| 2 ml NH4OH (27-29% concentrate) |
| 100 ml 2% NH4OH |

| **1% Citric Acid** |
| --- |
| 100 ml dH20 |
| 1.0 g Citric Acid |
| 100 ml 1% Citric Acid |

| **4.99pH Acetate Buffer Stock** |
| --- |
| 60 ml 1M Acetic Acid |
| 140 ml 1M Sodium Acetate |
| 200 ml 4.99 pH Acetate Buffer Stock |

| **4.99pH Acetate Buffer Working Solution** |
| --- |
| 238 ml dH2O |
| 12 ml 4.99 pH Buffer |
| 250 ml 4.99 pH Acetate Buffer Working Solution |

| **0.5% Sodium Thiosulfate** |
| --- |
| 400 ml dH2O |
| 2 g Sodium Thiosulfate |
| 400 ml 0.5% Sodium Thiosulfate |

| **Physical Developer** |
| --- |
| **Solution A** |
| 500 ml dH2O |
| 25 g Na2CO3 |
| 500 ml Solution A |
| **Solution B** |
| 500 ml dH2O |
| 1.0 g NH4NO3 |
| 1.0 g AgNO3 |
| 5.0 g Tungstosilicic Acid |
| 500 ml Solution B |
| **Solution C** |
| 250 ml dH2O |
| 0.5 g NH4NO3 |
| 0.5 g AgNO3 |
| 2.5 g Tungstosilicic Acid |
| 1.75 ml 37% Formaldehyde |
| 250 ml Solution C |

| **Physical Developer ABC** |  |
| --- | --- |
| Solution A | 100 ml |
| Solution B | 80 ml |
| Solution C | 20 ml |
